# Childhood Memories in Eating Disorders: An Explorative Study Using Diagnostic Imagery

**DOI:** 10.3389/fpsyg.2021.685194

**Published:** 2021-07-22

**Authors:** Barbara Basile, Chiara Novello, Simona Calugi, Riccardo Dalle Grave, Francesco Mancini

**Affiliations:** ^1^Association of Cognitive Psychology (APC), School of Cognitive Psychotherapy (SPC), Rome, Italy; ^2^Clinical Practice, Vicenza, Italy; ^3^Department of Eating and Weight Disorders, Villa Garda Hospital, Verona, Italy; ^4^Department of Human Sciences, Guglielmo Marconi University, Rome, Italy

**Keywords:** diagnostic imagery, eating disorders, anorexia nervosa, bulimia nervosa, early maladaptive schemas, parental schemas, unmet core needs

## Abstract

Together with socio-cultural components, the family environment and early parent–child interactions play a role in the development of eating disorders. The aim of this study was to explore the nature of early parent–daughter relationships in a sample of 49 female inpatients with an eating disorder. To acquire a detailed image description of the childhood experiences of the patient, we used diagnostic imagery, a schema therapy-derived experiential technique. This procedure allows exploring specific contents within the childhood memory (i.e., emotions and unmet core needs), bypassing rational control, commonly active during direct verbal questioning. Additionally, patients completed self-report measures to assess for eating disorder severity, general psychopathology, and individual and parental schemas pervasiveness. Finally, we explored possible differences in the diagnostic imagery content and self-report measures in two subgroups of patients with anorexia nervosa and bulimia nervosa. The results showed that the most frequently reported unmet needs within the childhood memories of patients were those of safety/protection, care/nurturance, and emotional expression, referred specifically to the maternal figure. Overall, mothers were described as more abandoning, but at the same time particularly enmeshed in the relationship with their daughters. Conversely, patients perceived their fathers as more emotionally inhibited and neglecting. Imagery-based techniques might represent a powerful tool to explore the nature of early life experiences in eating disorders, allowing a more detailed case conceptualization and addressing intervention on early-life vulnerability aspects in disorder treatment.

## Introduction

The nature of the association between parenting difficulties and eating disorder psychopathology is unclear, and the findings of the studies are still inconclusive. Literature exploring the connection between eating disorders and early parent–child relations includes studies based on the attachment theory and the parental bonding construct (Ward et al., 2000; Zachrisson and Skårderud, 2010). Insecure attachment (dismissed or preoccupied types) has been reported among individuals with eating disorders (Zachrisson and Skårderud, 2010). In particular, patients with anorexia nervosa restricting type display more often an avoidant attachment style, while individuals with bulimia nervosa more often report a preoccupied attachment (Zachrisson and Skårderud, 2010). Moreover, higher attachment anxiety has also been significantly related to greater eating disorder severity and poorer treatment outcomes (Illing et al., 2010). Other studies investigated parental bonding, specifically the two fundamental dimensions of care and protection. Overall, the results revealed that patients with eating disorders tend to report lower paternal care and perceived absent or weak bonding, with an overprotective mother style (for a review, see Tetley et al., 2014). However, the results were often conflicting due to the studied population (clinical or non-clinical groups), the eating disorder diagnostic subtype (Balottin et al., 2017), the treatment setting (i.e., hospitalization or outpatients treatment), and the severity of the disorder, which might be indicative of eventual comorbidities or specific personality features (Meneguzzo et al., 2021). Another branch of studies has shown the impact of traumatic experiences (i.e., physical, sexual, or emotional abuse) in the development and maintenance of an eating disorder (Dalle Grave et al., 1996; Backholm et al., 2013; Monteleone et al., 2020; Meneguzzo et al., 2021; Scharff et al., 2021), with higher levels of childhood trauma and a number of experienced traumatic episodes, being associated with more severe symptoms of eating disorders (Guillaume et al., 2016). Furthermore, early exposure to traumatic events might have an impact on the neurobiological development of the brain, specifically on the long-lasting neuroendocrine modifications that might account for a major risk of developing a future eating disorder (for a review, see Marciello et al., 2020).

Negative mental images characterize many psychological disorders (Brewin et al., 2010), and negative images about shape, weight, the self, and others play a role in the maintenance of eating disorders (Cooper, 2011). Within the schema therapy model (Young et al., 2003), emotional–experiential techniques based on imagery are used by the clinician to explore the content of the early childhood memories of patients and associate such contents with their early maladaptive schemas (henceforth called “schemas”) and their parental (or other caregivers) interpersonal schemas. Schema therapy is an integrated approach (i.e., cognitive-behavioral therapy, Gestalt, attachment theory, and transactional analysis) that emphasizes the role of biographical aspects in the development of maladaptive psychological patterns, through traumatization in childhood and frustration of basic childhood needs. Treatment based on this model uses both the therapeutic relationship and emotional techniques to address such unmet core needs, in order to help the client to fulfill such needs in a more functional way. Change of unhealthy coping strategies, at the basis of dysfunctional behaviors, might help to promote more healthy patterns. Several studies have shown that treatment based on schema therapy is very effective for patients with personality disorders (Giesen-Bloo et al., 2006; Farrell et al., 2009; Nadort et al., 2009; Bamelis et al., 2014; Dickhaut and Arntz, 2014), and better results are also reported for chronic Axis I disorders (Cockram et al., 2010; Malogiannis et al., 2014; Renner et al., 2016; Thiel et al., 2016).

Imagery is a powerful technique that allows access to early memories, bypassing cognitive, and rational control, that are commonly active during direct verbal questioning (Hackman et al., 2011). Imagery exercises can be used for diagnostic purposes, to clarify the biographical origin of actual dysfunctional schemas and emotional problems, as well as related behavioral patterns. This exercise is known as diagnostic imagery, or imagery for assessment, and commonly starts from a current negative emotional situation. Another way to access significant early contents starts directly with the visualization of a past memory, with specific instructions on the emotional valence of the episode to be retrieved (i.e., positive, negative, or neutral). To our knowledge, no study has investigated early parent–child memories through diagnostic imagery in patients with eating disorders. Previous studies have applied Imagery Rescripting (IR, Arntz and Weertman, 1999) to early memories as a therapeutic tool in the treatment of clients with anorexia or bulimia nervosa (Ohanian, 2002; Cooper et al., 2007; Cooper, 2011; Deguè et al., 2019; Zhou and Wade, 2021) and binge-eating disorder (Deguè et al., 2019). IR aims to address and change the meaning of adverse childhood events and emotions within the past episode, principally by satisfying the emotional unmet needs of the child, through guided mental visualization. Two papers reported a single-case study (Ohanian, 2002; Cooper, 2011), while another study compared one single session of cognitive restructuring technique with IR, on an image of social rejection, in individuals with binge-eating disorder and bulimia nervosa. In another study (Cooper et al., 2007), authors used one session of IR to change the negative self-beliefs of patients with bulimia nervosa, through the early negative memories associated with these beliefs. Moreover, in a meta-analysis, rescripting general negative imagery was shown to decrease dysfunctional attitudes and core beliefs associated with eating disorders (Morina et al., 2017). One very recent study (Zhou and Wade, 2021) failed to find a higher effect of one IR exercise associated with treatment as usual (compared with TAU without imagery), in a sample of patients with anorexia nervosa or otherwise specified feeding and eating disorders. These results could be due to the small sample size, the timing of the imagery intervention, which may not have been appropriate for the selected patients, and a short 4-week follow-up measurement. Finally, another study investigated spontaneous images, associated with negative core beliefs, occurring immediately before having worries about eating, weight, or shape, in individuals with bulimia nervosa (Somerville and Cooper, 2007). The authors found that patients diagnosed with bulimia nervosa reported significantly more negative self-core beliefs (assessed through the downward arrow technique, starting from the negative image) than the “dieters” and “healthy non-dieting” control groups. Overall, these findings show some evidence on the feasibility of applying imagery exercises to individuals with eating disorders, proposing the use of IR as a therapeutic tool.

According to the above premises, the aims of this study were: (i) to explore the content of negative childhood episodes (i.e., emotions and unmet core needs) in a sample of inpatients with eating disorders, through diagnostic imagery exercises; (ii) to investigate whether specific unmet core needs reported in the memory were associated with the individual and parental schemas of patients, as assessed through schema therapy-related self-report measures; and (iii) to explore the differences between anorexia nervosa and bulimia nervosa in individual and parental maladaptive schemas and the content of the childhood memories.

## Materials and Methods

### Participants

Forty-nine patients (mean age 25.2 [8.2] years; age range 18–51 years; all Caucasian women) with eating disorders participated in the study. All were above 18 years of age and were consecutively admitted to the eating disorder inpatient unit of the Villa Garda Hospital of Northern Italy. The patients were referred from all over Italy by general practitioners or by eating disorder specialists of outpatients. Thirty-three patients were diagnosed with anorexia nervosa (mean age 25.2 [8.7] years, BMI = 15.1), and 16 patients were diagnosed with bulimia nervosa (mean age 25.5 [7.04] years, BMI = 21.7). DSM-5 diagnosis was performed by an expert in eating disorders (RDG) using the Eating Disorder Examination (EDE) interview (Italian version) (Calugi et al., 2016). All the patients did not improve with less intensive treatment (e.g., outpatient treatment) or had an eating disorder of clinical severity not manageable in an outpatient setting. Patients with active substance misuse or any psychotic symptoms were not included in the study. Psychotropic medications were not prescribed during the treatment, and the psychotropic drugs being taken by patients at admission were gradually phased out during the first 2 weeks of hospitalization, while patients were under the supervision of clinic staff. The research was reviewed and approved by the Guglielmo Marconi University in Rome (Italy), and all participants gave written informed consent.

### Measures

The assessment took place on the first day of inpatient admission. Data collection included weight and height measurement, a face-to-face structured eating disorder diagnostic interview, and a package of questionnaires to evaluate eating disorder and general psychopathology. Patients were dressed in underwear without shoes. The body mass index (BMI) was derived by dividing the weight (in kilograms)/height squared (in meters).

The Eating Disorders Examination 17.OD (EDE Italian versions; Fairburn et al., 2009; Calugi et al., 2015) was used to evaluate the eating disorder psychopathology and to elicit the diagnosis of the eating disorder. A senior specialist in the field (RDG) completed the interview.

The Eating Disorders Examination Questionnaire (EDE-Q Italian version; Fairburn et al., 2009; Calugi et al., 2016) is a self-report version of the EDE. It is a 28-item self-report questionnaire that focuses on the patient report of symptom occurrence over the past 28 days and includes four subscales: “restraint concerns,” “eating concerns,” “weight concerns,” and “shape concerns.” The higher the score, the greater the eating disorder psychopathology reported by the patient. The Italian version showed good internal consistency and the test–retest reliability (Calugi et al., 2016).

The Brief Symptom Inventory (BSI; Derogatis and Melisaratos, 1983; De Leo et al., 1993) is the shortened version of the Symptom Checklist (SCL-90-R) assessing based on the response of an individual to 53 items (e.g., feeling fearful and mind going blank) using the five-point Likert scale (0 = not at all, 4 = extremely). In this study, we used the Global Severity Index (GSI), i.e., calculated as the mean of 53 items, and the BSI depression subscale, that is, calculated as the mean of 7 items, to evaluate the confounding role of depression in autobiographic memories (Dalgleish et al., 2003). The Italian version showed good internal consistency and the test–retest reliability (De Leo et al., 1993).

The Clinical Impairment Assessment Questionnaire (CIA Italian version; Bohn et al., 2008; Calugi et al., 2018) is a 16-item self-report measure of the severity of psychosocial impairment due to eating disorder features. It focuses on the past 28 days. The 16 items cover impairment in the domains of life that are typically affected by eating disorder psychopathology: mood and self-perception, cognitive functioning, interpersonal functioning, and work performance. The purpose of the CIA is to provide a simple single index of the severity of psychosocial impairment secondary to eating disorder features. The Italian validation showed good internal consistency and test–retest reliability (Calugi et al., 2018).

The Young Parenting Inventory (YPI Italian version; Young et al., 2003, 2007) is a self-report questionnaire assessing perceived parental experiences in youth (before the age of 11 years old). The YPI is designed to identify the parental origin of the schemas identified by Young et al. (2003). The questionnaire consists of 72 items, which break down into 17 parental schemas that are clustered into five domains. Respondents were asked to rate items about their experience of the attitude and behavior of their parents toward them on the six-point Likert scale (ranging from 1 = completely untrue to 6 = describes him/her perfectly). The final scoring of each schema was calculated in percentage (from 0% = schema is absent to 100% the schema is fully present). Two versions were administered: a version with statements about the mother and a version with statements about the father. The first domain, disconnection/rejection, refers to the expectation that parents should provide stability, security, and empathy and consists of the schemas abandonment/instability, mistrust/abuse, emotional deprivation, and defectiveness/shame. The second domain is related to impaired autonomy and performance and refers to the perception that parents are overprotective and undermining the confidence of the child. It includes the schemas dependence/incompetence, vulnerability to harm or illness, enmeshment/undeveloped self, and failure. The third domain, impaired limits, refers to permissiveness and lack of direction by the parents and consists of two schemas: entitlement/grandiosity and insufficient self-control/self-discipline. The fourth domain is other-directedness. It includes subjugation, self-sacrifice, and approval-seeking/recognition-seeking and refers to the perception that emotional needs of parents are valued more than the unique needs of the child. Finally, the inhibition domain concerns a demanding and punitive parenting style in which performance and perfectionism predominate over pleasure. This domain includes negativity/pessimism, emotional inhibition, unrelenting standards/hyper-criticalness, and punitiveness. The higher the score in the schema, the greater its pervasiveness reported by the patient.

The Young Schema Questionnaire—Short Form 3 (YSQ-S3 Italian version; Young et al., 2005; Aloi et al., 2020) is a 90-item questionnaire that assesses 18 individual early maladaptive schemas (see the previous paragraph where the YPI is described, for a complete overview of schemas and domains). Each schema is assessed according to five items (i.e., statements such as “I haven't had someone to nurture me, share him/herself with me, or care deeply about everything that happens to me”) scoring on a 6-point scale ranging from 1 = completely untrue of me to 6 = describes me perfectly. Final scores for each schema range from 5 to 30. The higher the score in the schema, the greater its pervasiveness. The Italian validation of the YSQ-S3 showed good internal consistency and excellent test–retest reliability (Aloi et al., 2020).

### Procedure

Two certified schema therapists (BB and CN) who did not know the patients collected the diagnostic imagery interviews between May 2018 and September 2020. Imagery interviews took place within the first 5 weeks of hospitalization of individuals. The experiential exercise started with women closing their eyes and imaging themselves in a safe place. Then, they were asked to leave the situation and enter a positive and safe situation (the “safe place imagery” exercise), such as being at a peaceful beach, or any other personal safe place. Later, the therapist asked the patient to wipe the image away and to recall a childhood memory where she had experienced a negative emotion with one or both parents. The therapists, then, asked the patient to describe the selected event in detail, speaking in the present tense and in the first person. Particular attention was paid to experienced emotions and associated bodily sensations. When the emotion was clear and intense enough, the patient was asked to express the needs associated with the childhood situation (e.g., a need for nurture, safety, acceptance, emotional expression, realistic limits, and autonomy). In the final phase, the woman was asked to return to the safe place, and when a positive emotional state was re-established, the imagery exercise was concluded. In a final debriefing phase, the therapist and the patient together discussed the content of the childhood memory, to detect whether there could be any link with symptoms of actual eating disorders. Patients were instructed to speak out loudly about their experience within the imagery exercise in order to record the interview for later decoding. Each interview took place in one single session and lasted between 15 and 30 min. A complete description of the imagery exercise (adapted from de Haan et al., 2017) is reported in the Supplementary Material **(Appendix A)**.

### Data Analyses

Three independent judges (all well-experienced schema and CBT therapists, working at the Association of Cognitive Psychology and School of Cognitive Psychotherapy), who were blind to the aim of the study, separately classified the imagery exercises of patients, after training and initial supervision (held by BB). Judges classified the content of the episode according to a grid with specific categories (age of the patient within the memory, the parent/s involved in the episode, its content, emotions, and unmet core needs within the episode, as referred by the patient, de Haan et al., 2017). Categories with all observations were included in the final scoring system. Data were analyzed using the Statistical Package for the Social Sciences, version 20.0 (SPSS Inc., Chicago, IL, United States).

Descriptive statistics included means and SDs, frequencies, and percentages for continuous (age, BMI, and self-report measures) and categorical (contents of the memory) variables. Partial correlations, controlling for BMI and BSI depression subscale score, to assess for the association between self-reported parental and individual schemas, and psychopathology measures were run. Moreover, the chi-square test was used to explore the most frequently reported emotions and unmet core needs within the childhood memory and the caregiver involved in the episode. Finally, partial correlations, controlling for BMI and BSI depression subscale score, between unmet core needs within the memory, and parental and individual schemas were analyzed. As well, linear regression analysis was run to assess the extent to which unmet core needs in the past (IV) were associated with the current self-reported parental (DV) and individual schemas (DV).

Differences between patients with anorexia nervosa and bulimia nervosa in the self-report measures (i.e., EDE-Q, CIA, BSI, YPI, and YSQ global and subscale scores) and the content of the imagery exercises (i.e., age within the memory, parent involved in the episode, emotions, and unmet core needs) were explored through Mann-Whitney *U*-test and chi-square test, respectively. To avoid type I errors, the *p*-value for comparison tests was set at 0.05/18 = 0.003. Logistic regression was performed to test the extent to which emotions (IV) and unmet core needs (IV) of childhood memory were associated with the belongingness of participants to the specific group of ED (AN or BN, DV).

## Results

### Whole Sample Analysis

Demographical information, severity of the eating disorder, and general psychopathology levels are reported in Table 1. Means, SDs, and scoring ranges in parental (YPI) and individual (YSQ) schemas are shown in Table 2. Differences in parental schemas (YPI mother vs. YPI father version) were determined through two-tailed Wilcoxon tests (see Table 2, for statistical significances). Partial correlation analyses, controlling for BMI and levels of depression, showed some significant correlations between the parental (YPI) and individual (YSQ) schemas of patients (see Table 3).

**Table 1:** Clinical data, eating disorder, and general psychopathology by eating disorder diagnosis.

	**Total eating disorder sample *n* = 49 Mean (*SD*) [range]**	**Anorexia nervosa *n* = 33 Mean (*SD*) [range]**	**Bulimia nervosa *n* = 16 Mean (*SD*) [range]**	***p***
Age in years	25.44 (8.22) [18–51]	25.28 (8.77) [18–51]	25.58 (7.04) [18–40]	0.464
BMI	17.07 (4) [9.7–29.4]	15.07 (3.96) [9.7–18.11]	21.74 (2.74) [18.8–29.3]	<0.001
Days of hospitalization	29.38 (16.08) [0–68]	30.55 (14.00) [0–61]	27.71 (20.74) [0–68]	0.282
Years of formal education	13.94 (2.41) [8–18]	14.25 (2.26) [12–18]	13.2 (2.65) [8–18]	0.381
Global score (EDE-Q)	4.3 (1.25) [0.79–5.95]	4.21 (1.18) [0.91–5.70]	4.5 (1.34) [0.79–5.95]	0.216
Restraint eating (EDE-Q)	3.94 (1.66) [0–6]	4.27 (1.51) [0–6]	3.55 (1.84) [1.20–6]	0.174
Eating concern (EDE-Q)	3.80 (1.47) [0.67–6]	3.71 (1.36) [0.67–6]	4.81 (1.52) [1.2–6]	0.053
Weight concern (EDE-Q)	4.28 (1.62) [0.8–6]	4.25 (1.55) [0.8–6]	4.9 (1.51) [1.2–6]	0.252
Shape Concern (EDE-Q)	4.66 (1.45) [0.5–6]	4.62 (1.37) [0.5–6]	5.01 (1.50) [0.75–6]	0.125
BSI	2.28 (0.76) [0.42–4.6]	2.29 (0.75) [0.62–3.47]	2.25 (0.82) [0.4–4.6]	0.466
Depression (BSI)	2.72 (0.91) [0.71–4]	2.62 (0.87) [0.71–4]	2.99 (1.00) [0.71–4]	0.242
CIA	37.73 (9.41) [8–48]	37.35 (9.15) [8–48]	38.6 (10.25) [17–48]	0.957

*Data are presented as mean (SD)*.

*BMI, body mass index; BN, bulimia nervosa; BSI, Brief Symptoms Inventory; CIA, Clinical Impairment Assessment; EDE-Q, Eating Disorders Examination Questionnaire; SD, standard deviation*.

**Table 2:** Means and SD in individual and parental schemas in eating disorders are reported.

**Schemas in eating disorders**	**Individual Mean (*SD*) [range]**	**Mother Mean (*SD*) [range]**	**Father Mean (*SD*) [range]**	**Wilcoxon**		**Schemas in eating disorders**	**Individual Mean (*SD*) [range]**	**Mother Mean (*SD*) [range]**	**Father Mean (*SD*) [range]**	**Wilcoxon**	
				***Z***	***P***					***Z***	***P***
Emotional deprivation	12.2 (5.1) [5–29]	12 (20.1) [0–100]	29.1 (36.1) [0–100]	−3.25	0.001	Self-sacrifice	19.6 (5.3) [6–30]	27.1 (18.4) [0–100]	18.2 (15.4) [0–75]	−2.77	0.006
Abandonment	18.7 (6.4) [6–30]	3.1 (8.3) [0–25]	2 (8.6) [0–25]	−3.07	0.002	Subjugation	16.2 (6.3) [6–29]	6.7 (17.6) [0–75]	4.1 (12.9) [0–50]	−1.15	0.24
Abuse/mistrust	16.8 (6.0) [5–29]	1.5 (7.9) [0–50]	0.5 (3.6) [0–25]	−0.81	0.41	Admiration and approval seeking	17.7 (6.3) [5–28]	10.4 (25.1) [0–100]	8.3 (18.8) [0–100]	−3.29	0.74
Defectiveness/shame	18.2 (8.0) [5–30]	5.7 (18.7) [0–100]	5.7 (17.2) [0–100]	0	1	Pessimism/negativism	17.6 (5.5) [6–29]	18.2 (24) [0–100]	14 (19.9) [0–75]	−1.03	0.30
Social isolation	18.4 (6.8) [5–30]	–	–	–	–	Emotional inhibition	14.9 (5.9) [5–30]	21.5 (27.1) [0–80]	38.7 (27.1) [0–100]	−3.45	0.001
Dependency/incompetency	14.1 (6.4) [5–26]	13.8 (27.3) [0–100]	8.3 (22.3) [0–100]	−1.22	0.21	Unrelenting standards	20.2 (4.5) [10–28]	13.8 (27.3) [0–100]	23.8 (29) [0–100]	−0.62	0.52
Vulnerability to harm and illness	12.2 (5.5) [4–24]	18.7 (25.5) [0–100]	9.3 (16.1) [0–50]	−2.81	0.005	Punitiveness	18.6 (6.4) [4–30]	13.5 (24.1) [0–75]	14 (27.2) [0–100]	−0.027	0.97
Enmeshment/undeveloped self	13.6 (5.5) [5–24]	25 (27.2) [0–75]	10.2 (14.8) [0–50]	−3.85	0.000	Insufficient self-control	16.7 (5.4) [5–28]	6.2 (15) [0–75]	6.7 (15.2) [0–75]	−3.78	0.70
Failure	17.8 (8.0) [6–30]	0.5 (3.6) [0–25]	1.5 (6.1) [0–25]	−1.41	0.15	Entitlement/grandiosity	14.6 (4.5) [7–26]	20.3 (23.4) [0–75]	16.1 (22.1) [0–75]	−1.88	0.06

*Differences between parental schemas (YPI mother vs. father version) were calculated through the two-tailed Wilcoxon tests*.

**Table 3:** Partial correlations measuring the association between parental and individuals schemas, controlling for BMI and depression, are reported.

	***M* (*SD*)**	**1**	**2**	**3**	**4**	**5**	**6**	**7**	**8**	**9**	**10**	**11**	**12**	**13**	**14**	**15**	**16**
1. Maternal emotional deprivation	12.1 (20.1)	–															
2. Maternal abandonment schema	3.1 (8.3%)	0.20	–														
3. Maternal enmeshment/undeveloped self-schema	25 (27.2)	**−0.28^°^**	0.16	–													
4. Maternal subjugation schema	6.7 (17.6)	0.08	−0.06	**0.38^°°^**	–												
5. Maternal punishment schema	13.5 (24.1)	0.17	**0.30^°^**	0.48^**^	0.67^**^	–											
6. Maternal unrelenting standards schema	26.7 (30.1)	0.18	0.10	0.36^*^	0.64^**^	0.62^**^	–										
7. Paternal submission schema	4.1 (12.9)	0.35^*^	−0.14	0.07	0.57^**^	0.50^**^	**0.29^°^**	–									
8. Paternal dependency/incompetency schema	8.3 (22.3)	0.04	0.02	−0.09	0.25	0.23	0.17	**0.31^°^**	–								
9. Individual emotional deprivation schema	12.1 (5.1)	**0.41^°°^**	**0.29^°^**	−0.07	0.05	0.15	0.19	0.09	−0.16	–							
10. Individual abandonment schema	18.5 (6.4)	**0.34^°^**	0.06	−0.03	−0.15	−0.16	−0.28	−0.08	−0.15	**0.29^°^**	–						
11. Individual abuse/mistrust schema	16.6 (6)	**0.30^°^**	0.04	−0.26	−0.25	−0.20	−0.13	−0.02	−0.17	**0.38^°°^**	0.56^**^	–					
12. Individual social isolation schema	18.3 (6.8)	**0.29^°^**	−0.02	−0.08	−0.17	−0.07	0.07	0.03	−0.13	0.55^**^	0.53^**^	0.45^**^	–				
13. Individual dependency/incompetency schema	14 (6.4)	**0.29^°^**	0.13	0.01	0.08	0.20	0.24	0.27	0.15	0.47^**^	0.47^**^	**0.26**	0.60^**^	–			
14. Individual subjugation schema	16 (6.2)	0.36^*^	0.13	0.16	**0.31^°^**	0.25	0.26	0.37^*^	0.17	0.36^*^	0.54^**^	**0.41^°°^**	**0.42^°°^**	0.68^**^	–		
15. Individual enmeshment/undeveloped self-schema	13.4 (5.4)	−0.11	0.08	0.62^**^	0.44^**^	**0.43^°°^**	**0.33^°^**	**0.30^°^**	0.06	0.19	−0.01	−0.09	0.10	**0.33^°^**	0.49^**^	–	
16. Individual insufficient self-control schema	16.7 (5.5)	**0.31^°^**	0.34^*^	−0.04	−0.07	0.04	**0.28^°^**	−0.05	**−0.31^°^**	**0.33^°^**	**0.41^°°^**	0.27	**0.43^°°^**	0.56^**^	**0.29^°^**	−0.05	–
17. Individual unrelenting standards schema	20.1 (4.5)	0.24	−0.16	0.13	0.19	0.10	0.23	0.36^*^	0.25	0.18	0.09	0.18	**0.31^°^**	0.16	**0.41^°°^**	0.25	−0.08

*BMI, body mass index. Statistically significant associations are reported in bold*.

° *p < 0.05*,

°° *p < 0.005*,

* *p < 0.01, and*

** *p < 0.001*.

#### Diagnostic Imagery Interview

According to the information collected through the imagery exercises, we analyzed the content of the past memories examining specific categories. The mean age of patients within the childhood episode was approximately 9.3 (*SD* = 3.11, range from 3 to 14) years, whereas the caregivers involved in the memory were the mother (*n* = 21, 43%), the father (*n* = 11, 22%), and both parents together (*n* = 15, 31%), with two (4%) patients reporting significant others to be involved. The most frequently reported emotions within the memory included (participants reported a maximum of three emotions within the episode), in order, sadness equally as often as fear/anxiety (*n* = 24, 27.2%), anger (*n* = 13, 14%), shame equally as often as guilt (*n* = 9, 10.2%), surprise (*n* = 4, 4.5%), emptiness and humiliation (*n* = 2, 2.2% each), and self-disgust (*n* = 1, 1%). The unmet core needs within the episode (in a range of 1–3 needs per episode) included safety and protection (*n* = 27, 29.5%); care, nurturance, and safe attachment (*n* = 22, 24%); emotional expression (*n* = 13, 14%), attention (*n* = 11, 12%), reassurance (*n* = 6, 6.5%), empathy and acceptance (*n* = 5, 5.5%), limit setting (*n* = 1, 1%), and play (*n* = 1, 1%). Due to the low number of cases in some categories, we will analyze only those classes including more than 10 cases. The negative emotions and unmet needs reported in the memory referred to contents such as physical or emotional abuse (i.e., parents fighting against each other or against the patient as a child), abandonment (i.e., parent leaving the child or threatening her about doing so), and inhibition of the feelings of patient (i.e., the child inhibits and hides her negative feelings to avoid the suffering of parent or his/her negative reaction).

Partial correlations (controlling for BMI and BSI depression subscale score) between emotions and unmet core needs within the memory, and core needs and parental and individual schemas were analyzed. The lack of safety and protection need was positively associated with emotions of fear and anxiety (*r* = 0.45, *p* = 0.001) and negatively correlated with feelings of sadness (*r* = −0.31, *p* = 0.02). Likewise, the need for care, nurturance, and attachment was inversely correlated with the emotions of sadness (*r* = −0.36, *p* = 0.01) and shame (*r* = −0.33, *p* = 0.01), whereas the unmet core need for attention was positively associated with feelings of sadness (*r* = 0.43, *p* = 0.003). When considering the associations between unmet core needs within the early memory and actual schemas, significant correlations were observed between the need for safety and protection, and emotional deprivation (*r* = 0.31, *p* = 0.03) and failure (*r* = 0.33, *p* = 0.02) schemas of patients, and maternal (*r* = 0.43, *p* = 0.003) and paternal (*r* = 0.33, *p* = 0.02) emotional deprivation and paternal emotional inhibit_on (*r* = −0.33, *p* = 0.02) schemas of patients. The need for care,-nurturance, and attachment correlated with paternal pessimism/negativism (*r* = 0.35, *p* = 0.01) and dependency (*r* = 0.29, *p* = 0.05) schemas; whereas the need for emotional expression correlated with paternal (*r* = −0.32, *p* = 0.03) emotional deprivation schema.

Multiple linear regression analyses were run to assess the extent to which unmet core needs (IV) in the past memory were significantly associated with current self-reported parental (DV) and individual schemas (DV). The need for safety and protection was significantly associated with maternal [*R*^2^ = 0.17, *F*_(1,46)_ = 9.56, *p* = 0.003, β = 0.41, *p* < 0.005] and emotional deprivation schema of patients [*R*^2^ = 0.08, *F*_(2,48)_ = 4.34, *p* = 0.04; β = 0.29, *p* < 0.05]. The need for care, nurturance, and attachment was significantly associated with maternal vulnerability to harm and illness [*R*^2^ = 0.12, *F*_(2,46)_ = 3.21, *p* = 0.05]. While entering personal schemas as a dependent variable, this unmet core need within the childhood memory was significantly associated with mistrust/abuse [*R*^2^ = 0.32, *F*_(6,42)_ = 4.42, *p* = 0.008; β = −0.68, *p* < 0.001], inadequacy/shame (β = 0.56, *p* < 0.05), and failure (β = −0.51, *p* < 0.05) schemas. The need for emotional expression was significantly associated with maternal emotional deprivation [*R*^2^ = 0.10, *F*_(1,46)_ = 5.36, *p* = 0.025; β = −0.32, *p* < 0.05] and with paternal schemas [*R*^2^ = 0.33, *F*_(3,44)_ = 7.32, *p* = 0.000] of emotional deprivation (β = −0.30, *p* < 0.05), punishment (β = −0.41, *p* < 0.005), and search for approval and admiration (β = 0.33, *p* < 0.05). Finally, the need for attention was significantly associated with paternal schemas [*R*^2^ = 0.17, *F*_(2,45)_ = 4.62, *p* = 0.015] of enmeshment/undeveloped self (β = −0.33, *p* < 0.05) and emotional deprivation (β = 0.29, *p* < 0.05).

Finally, we ran some partial correlations (controlling for BMI and BSI depression subscale score) between the severity of the eating disorder, general levels of psychopathology, psychosocial impairment, and individual and parental schemas. No significant associations were detected between clinical measures (i.e., EDE-Q, BSI, and CIA) and individual schemas. The EDE-Q total score significantly correlated with paternal vulnerability to harm schema (*r* = 0.32, *p* = 0.03). General psychopathological severity of symptoms (BSI) was positively associated with paternal vulnerability to harm (*r* = 0.33, *p* = 0.02) and defectiveness/shame (*r* = 0.34, *p* = 0.02) schemas. Psychosocial impairment (CIA) significantly correlated with maternal entitlement/grandiosity (*r* = −0.35, *p* = 0.02) and enmeshment (*r* = −0.31, *p* = 0.04) schemas, and paternal enmeshment (*r* = −0.36, *p* = 0.01) and emotional inhibition (*r* = −0.41, *p* = 0.006) schemas.

### Within-Group Differences

Non-parametric analyses were executed to detect differences between patients with anorexia nervosa and bulimia nervosa (see Table 1). When considering group differences within parental schemas (YPI), only the insufficient self-control paternal schema resulted to be significantly more pervasive in patients with bulimia nervosa than in those with anorexia nervosa (*M* = 2.27, DS = 7.29 in AN; *M* = 16.66, DS = 22.49 in BN, *Z* = −3.02, *p* = 0.003). When considering the schemas of patients (YSQ), again, only the insufficient self-control schema was significantly more pervasive in patients with bulimia nervosa (*M* = 20.13, DS = 5.57) than in those with anorexia nervosa (*M* = 15.32, DS = 54.79; *Z* = −2.97, *p* = 0.003).

#### Diagnostic Imagery Interview

No significant differences were observed between the two eating disorder groups of patients when examining childhood age within the memory, caregiver, emotions, and unmet core need reported in the imagery exercises. We ran a logistic regression analysis to detect whether specific core needs within the childhood memory would be associated with the belongingness to the eating disorders diagnosis. The overall logistic regression model (Nagelkerke *R*^2^ = 0.23, *p* < 0.05) revealed that the unmet need of care, nurturance, and attachment was expressively coupled with the diagnosis of bulimia nervosa [*B* = 2.03, SE = 0.73, Wald = 7.63, *p* < 0.05, Exp(*B*) = 7.66, 95% CI = 1.08, 32.51] and not with the diagnosis of anorexia nervosa.

## Discussion

In this study, we used diagnostic imagery, an emotional experiential technique derived from the schema therapy model (Young et al., 2003), to investigate the content of negative early childhood memories involving parental figures in a sample of female inpatients with eating disorders. We focused specifically on the negative emotions and the related unmet core needs within the past memory. Furthermore, we investigated the pervasiveness of individual and parental schemas of patients and their possible association with the unmet core needs in the childhood episode. Finally, differences between individuals diagnosed with anorexia nervosa and bulimia nervosa were explored.

When exploring the content of the negative early episodes, we found that the reported overall mean age of the child was around 9 years old and that the mother was the most frequently reported parental figure being involved in the negative childhood memory. The most recurrently reported emotions were fear and anxiety, which were congruently related to the unmet core need of safety and protection. The typical contents associated with this need were related to physical abuse toward the patient as a child or parents fighting against each other in the presence of their daughter. This need is typically frustrated when physical, sexual, or emotional abuse takes place and seems specifically associated with the mistrust/abuse schema (Young et al., 2003). Within our sample, the early unmet need for safety and protection was positively associated with emotional deprivation and paternal emotional inhibition schemas of both parents and with emotional deprivation and failure schemas of patients. The second most frequent unmet core need within the memory was lack of care, nurturance, and safe attachment. These needs were more often reported in those episodes where one or both parents were unable to take care of the child, abandoning her or simply being unable to cope with the negative situation. It covers the need of the child for support and care by caregivers and is commonly fulfilled through the presence of a stable and predictable emotional attachment figure, which reassures, guides, and takes care of the child. Not meeting the need of the child might foretell the development of an abandonment and instability schema (Young et al., 2003). We found this need to be inversely correlated with feelings of sadness and shame within the childhood episode and with paternal pessimism/negativism and dependency schemas. The third most frequently reported unmet need within the childhood memory was emotional expression. This need is unmet when spontaneous manifestations are mocked, punished or ignored, and when needs or normative or social rules of others are considered more important than that of the child. According to schema therapy, not meeting the need of emotional expression might lead to the development of the emotional inhibition maladaptive schema (Young et al., 2003). Emotional expression inhibition within the early memory correlated with paternal emotional deprivation schema.

We also observed differences in reported the pervasiveness of parental schemas, where mothers were described as more enmeshed, abandoning, vulnerable to harm and illness, and more self-sacrificing than fathers. Instead, our patients described their fathers as being more neglecting and emotionally inhibiting than their mothers. Moreover, individual schema of enmeshment of patients correlated with maternal enmeshment, subjugation, and punishment schemas. These findings seem in contrast to the parental overprotection perceived by patients with an eating disorder described by other authors (Tetley et al., 2014). Enmeshment and vulnerability to harm might represent a dysfunctional way to feel protected, limiting the independence and autonomy of the child. In a previous study (Sheffield et al., 2009), maternal emotional inhibition was related to the body dissatisfaction of patients, with the mediation of a behavioral-somatic avoidance process. Also, both parental figures were equally described as higher in emotional deprivation, overprotection (i.e., inhibition of fostering the independency of the child), belittling behaviors, punitiveness, control, and emotional inhibition in patients, compared with a non-clinical healthy sample. Another recent similar study (Basile et al., 2020) observed a higher inhibition schema only in the paternal figure in individuals with an eating disorder compared with a non-clinical group.

We also considered the association between psychopathology severity and pervasiveness of schemas. Higher levels of eating disorders and general psychopathology significantly correlated with paternal vulnerability to harm schema, with higher levels of general dysfunction being also associated with paternal defectiveness/shame schemas. Further, higher severity of psychosocial impairment due to eating disorder was associated with more pervasive maternal entitlement/grandiosity and enmeshment schemas, and with paternal enmeshment and emotional inhibition schemas. Conversely, we did not detect any significant association between psychopathology and psychosocial impairment and schemas of patients.

When exploring for potential differences in the content of the childhood memories and self-reported schemas between the two eating disorder categories, we observed that patients with bulimia nervosa reported more pervasive individual and paternal insufficient self-control schemas than patients with anorexia nervosa. Indeed, binge-eating episodes are commonly characterized by a feeling of being out of control during the eating episode. According to an emotion regulation explanation, they are not only the effect of dietary restraint but also of an avoidant self-soother coping mode (Luck et al., 2006; Waller et al., 2007; Brown et al., 2016; Simpson and Smith, 2019). The lack of self-control correlated with maternal emotional deprivation, abandonment, and unrelenting standards/ hypercriticism schemas. A previous study detected specific schema profiles in populations with an eating disorder, being characterized by critical, punitive, and demanding parental schema modes (Simpson and Smith, 2019). These schema modes refer to the underlying unrelenting standards and hypercriticism schema that is characterized by high and unrealistic expectations of perfectionism, where severe criticism and punishment might follow potential mistakes or not-accomplished standards. Coherently, some studies have observed an association between parental expectations of perfectionism and achievement and an increased risk for eating disorders in their children (e.g., Woodside et al., 2002; Luca, 2010). No differences between subgroups were observed in the content of the childhood memories, although the early unmet need of care, nurturance, and safe attachment of patients was significantly associated with the diagnosis of bulimia nervosa, but not with the diagnosis of anorexia nervosa.

This study has several limitations. First, because of its qualitative nature, the content of the childhood memories might be inaccurately retrieved as the original memory might have been modified during recall. Also, one single memory cannot be representative of the nature of the relationship between the caregivers and the child. Second, as we have also used self-report measures, patients might have distorted and not adequately represented the earlier parental style. In line with this, as it is a cross-sectional study, we cannot make any causal inference between the contents within the childhood memory and self-report measures. Longitudinal studies, like that of Zubatsky et al. (2015), should be considered to make these predictions. Third, the absence of different clinical and non-clinical control groups does not permit to assess if our findings are specific to patients with an eating disorder. Furthermore, the exploratory nature of the study with the inclusion of a convenience sample of patients suffering from severe eating disorder psychopathology and a small sample size of patients with bulimia nervosa compromises the generalizability of the findings. Finally, our data cannot be generalized to patients with other specified or unspecified feeding and eating disorder diagnoses.

In summary, our study suggests that individuals with an eating disorder undergoing diagnostic imagery recalled more often negative early memories involving the maternal figure, mainly reporting unmet needs related to safety and protection, care and nurturance, and emotional inhibition. Patients further described their mothers as more abandoning, but at the same time more enmeshed, in the relationship with them, than the fathers. Conversely, paternal figures were perceived as more emotionally inhibited and neglecting than mothers. Diagnostic imagery is a short and feasible technique that could be used in the early phases of treatment of eating disorders to identify specific parent–child interactions and the unmet core needs of patients within a relationship. These contents might be further explored during treatment to address specific maladaptive schemas and their association with dysfunctional coping strategies of patients (i.e., dietary restriction, hyper-control, and perfectionistic behaviors). Further, in a later phase of treatment, these early memories could be addressed through Imagery Rescripting technique, allowing such needs to be fulfilled through imagination. Future studies should assess whether specific individual or parental schemas, as considered by the schema therapy approach, could somehow predict treatment outcomes. If this were the case, imagery assessment might become a useful clinical tool to assess eating disorders psychopathology, to develop a case conceptualization, or to inform directions for treatment.

## Data Availability Statement

The raw data supporting the conclusions of this article will be made available by the authors, without undue reservation.

## Ethics Statement

The studies involving human participants were reviewed and approved by Università Guglielmo Marconi—Roma. The patients/participants provided their written informed consent to participate in this study.

## Author Contributions

BB and FM contributed equally to the conception and design of the study. BB and CN conducted the diagnostic imagery interviews and organized the database. RD conducted all the assessments. BB and SC performed the statistical analysis. BB wrote the first draft of the manuscript. All authors contributed to manuscript revision, read, and approved the submitted version.

## Conflict of Interest

The authors declare that the research was conducted in the absence of any commercial or financial relationships that could be construed as a potential conflict of interest.
