# Supplementary material for: Childhood Memories in Eating Disorders: An Explorative Study Using Diagnostic Imagery
Source: Front Psychol. 2021 Jul 22;12:685194. doi: 10.3389/fpsyg.2021.685194 (PMC8339314; doi:10.3389/fpsyg.2021.685194)
Supplement: Supplementary file 1 [file Table_1.DOCX]

***Supplementary Material***

**Appendix A**

***Safe Place Imagery***

Instructions:

“*Please put yourself in a comfortable position and close your eyes…*

*Try to recall an image of a place that is safe for you… You can be alone or with someone.*

*The important thing is that you visualize a place where you can feel completely safe and at your ease. Please describe the details, the colours, the sounds, smells and bodily sensation, trying to make the image as vivid as possible*”.

***Diagnostic imagery***

Instructions:

1. *“Now please let this safe place image fade away and, always keeping your eyes closed, try to get an image of your self as a child where you were with one or both your parents in a negative and painful situation.*
2. *Please tell me what happens using the present tens, talking from the perspective of you as a child who is going through this situation.”*

Specific questions:

1. *How old are you in the image?*
2. *Where are you? Tell me what happens.*
3. *Tell me what you see, hear, feel and, smell*
4. *What do you feel (emotionally?)*
5. *Where (in your body) do you feel this? (*or, if patient has difficulties with staying in the child perspective: “*What does little (name of the patient) …. feel?” “Where does little …. feel this?”)*
6. *What do you think? (*or, if patient has difficulties with staying in the child perspective*: ”What does little …. think?”)*
7. *What do you need? (*or, if patient has difficulties with staying in the child perspective*: “What does little …. need?”).*
8. *Now please try to recall the image of your safe place. Please describe the details, the colours, the sounds, smells and bodily sensation, trying to make the image as vivid as possible”.*

Debriefing phase*.*
